# Identification of potential C1-binding sites in the immunoglobulin C_L_ domains

**DOI:** 10.1093/intimm/dxae017

**Published:** 2024-04-02

**Authors:** Saeko Yanaka, Atsuji Kodama, Shigetaka Nishiguchi, Rina Fujita, Jiana Shen, Pornthip Boonsri, Duckyong Sung, Yukiko Isono, Hirokazu Yagi, Yohei Miyanoiri, Takayuki Uchihashi, Koichi Kato

**Affiliations:** Department of Creative Research, Exploratory Research Center on Life and Living Systems (ExCELLS), Department of Life and Coordination-Complex Molecular Science, Institute for Molecular Science (IMS), National Institutes of Natural Sciences, 5-1 Higashiyama, Myodaiji, Okazaki 444-8787, Japan; Department of Structure Biology and Biomolecular Engineering, Faculty and Graduate School of Pharmaceutical Sciences, Nagoya City University, 3-1 Tanabe-dori, Mizuho-ku, Nagoya 467-8603, Japan; Department of Medicinal Sciences, Graduate School of Pharmaceutical Sciences, Kyushu University, 3-1-1 Maidashi, Higashi, Fukuoka 812-8582, Japan; Department of Creative Research, Exploratory Research Center on Life and Living Systems (ExCELLS), Department of Life and Coordination-Complex Molecular Science, Institute for Molecular Science (IMS), National Institutes of Natural Sciences, 5-1 Higashiyama, Myodaiji, Okazaki 444-8787, Japan; Department of Creative Research, Exploratory Research Center on Life and Living Systems (ExCELLS), Department of Life and Coordination-Complex Molecular Science, Institute for Molecular Science (IMS), National Institutes of Natural Sciences, 5-1 Higashiyama, Myodaiji, Okazaki 444-8787, Japan; Department of Creative Research, Exploratory Research Center on Life and Living Systems (ExCELLS), Department of Life and Coordination-Complex Molecular Science, Institute for Molecular Science (IMS), National Institutes of Natural Sciences, 5-1 Higashiyama, Myodaiji, Okazaki 444-8787, Japan; Department of Creative Research, Exploratory Research Center on Life and Living Systems (ExCELLS), Department of Life and Coordination-Complex Molecular Science, Institute for Molecular Science (IMS), National Institutes of Natural Sciences, 5-1 Higashiyama, Myodaiji, Okazaki 444-8787, Japan; Department of Structure Biology and Biomolecular Engineering, Faculty and Graduate School of Pharmaceutical Sciences, Nagoya City University, 3-1 Tanabe-dori, Mizuho-ku, Nagoya 467-8603, Japan; Department of Chemistry, Faculty of Science, Srinakharinwirot University, Bangkok 10110, Thailand; Department of Creative Research, Exploratory Research Center on Life and Living Systems (ExCELLS), Department of Life and Coordination-Complex Molecular Science, Institute for Molecular Science (IMS), National Institutes of Natural Sciences, 5-1 Higashiyama, Myodaiji, Okazaki 444-8787, Japan; Department of Structure Biology and Biomolecular Engineering, Faculty and Graduate School of Pharmaceutical Sciences, Nagoya City University, 3-1 Tanabe-dori, Mizuho-ku, Nagoya 467-8603, Japan; Department of Creative Research, Exploratory Research Center on Life and Living Systems (ExCELLS), Department of Life and Coordination-Complex Molecular Science, Institute for Molecular Science (IMS), National Institutes of Natural Sciences, 5-1 Higashiyama, Myodaiji, Okazaki 444-8787, Japan; Department of Creative Research, Exploratory Research Center on Life and Living Systems (ExCELLS), Department of Life and Coordination-Complex Molecular Science, Institute for Molecular Science (IMS), National Institutes of Natural Sciences, 5-1 Higashiyama, Myodaiji, Okazaki 444-8787, Japan; Department of Structure Biology and Biomolecular Engineering, Faculty and Graduate School of Pharmaceutical Sciences, Nagoya City University, 3-1 Tanabe-dori, Mizuho-ku, Nagoya 467-8603, Japan; Research Center for Next-Generation Protein Sciences, Institute for Protein Research, Osaka University, 3-2 Yamadaoka, Suita, Osaka 565-0871, Japan; Department of Creative Research, Exploratory Research Center on Life and Living Systems (ExCELLS), Department of Life and Coordination-Complex Molecular Science, Institute for Molecular Science (IMS), National Institutes of Natural Sciences, 5-1 Higashiyama, Myodaiji, Okazaki 444-8787, Japan; Department of Physics and Institute for Glyco-core Research (iGCORE), Nagoya University, Furocho, Chikusa, Nagoya, 464-8602 Aichi, Japan; Department of Creative Research, Exploratory Research Center on Life and Living Systems (ExCELLS), Department of Life and Coordination-Complex Molecular Science, Institute for Molecular Science (IMS), National Institutes of Natural Sciences, 5-1 Higashiyama, Myodaiji, Okazaki 444-8787, Japan; Department of Structure Biology and Biomolecular Engineering, Faculty and Graduate School of Pharmaceutical Sciences, Nagoya City University, 3-1 Tanabe-dori, Mizuho-ku, Nagoya 467-8603, Japan

**Keywords:** C1q, C_L_, high-speed atomic force microscopy, immunoglobulin G, nuclear magnetic resonance

## Abstract

Immunoglobulin G (IgG) molecules that bind antigens on the membrane of target cells spontaneously form hexameric rings, thus recruiting C1 to initiate the complement pathway. However, our previous report indicated that a mouse IgG mutant lacking the Cγ1 domain activates the pathway independently of antigen presence through its monomeric interaction with C1q via the C_L_ domain, as well as Fc. In this study, we investigated the potential interaction between C1q and human C_L_ isoforms. Quantitative single-molecule observations using high-speed atomic force microscopy revealed that human Cκ exhibited comparable C1q binding capabilities with its mouse counterpart, surpassing the Cλ types, which have a higher isoelectric point than the Cκ domains. Nuclear magnetic resonance and mutation experiments indicated that the human and mouse Cκ domains share a common primary binding site for C1q, centred on Glu194, a residue conserved in the Cκ domains but absent in the Cλ domains. Additionally, the Cγ1 domain, with its high isoelectric point, can cause electrostatic repulsion to the C1q head and impede the C1q-interaction adjustability of the Cκ domain in Fab. The removal of the Cγ1 domain is considered to eliminate these factors and thus promote Cκ interaction with C1q with the potential risk of uncontrolled activation of the complement pathway *in vivo* in the absence of antigen. However, this research underscores the presence of potential subsites in Fab for C1q binding, offering promising targets for antibody engineering to refine therapeutic antibody design.

## Teaser

Antigen-independent complement-binding sites in IgG C_L_ domains

## Introduction

Immunoglobulin G (IgG), a central player in the humoral immune system, functions as a hub mediating recognition of foreign antigens by the Fab portions and promotion of effector functions in the Fc portion. Each of the Fab and Fc portions is constituted from multiple Ig-fold domains. The Fab portion consists of two variable domains V_H_ and V_L_, and two constant domains C_L_ and Cγ1, while the Fc portion consists of two Cγ2 and two Cγ3 domains. Consequently, the conventional IgG structure is composed of twelve domains arranged in a Y-shaped quaternary structure, in which the Cγ1 and Cγ2 domains are connected through a flexible hinge segment (1, 2).

The V_H_ and V_L_ domains are directly involved in specific antigen binding, which triggers interactions of IgG with effector molecules, such as complement component C1 and Fcγ receptors (FcγRs), primarily at the hinge-proximal sites in the Cγ2 domains, leading to the elimination of foreign substances. The antigen-dependent promotion of effector functions is crucial in the immune system because their unchecked activation in the absence of antigens poses a risk of harm to the organism. The molecular mechanisms of such regulation are supposed to involve conformational changes and the molecular assembly of IgG initiated by antigen binding, which is exemplified by the antigen-dependent activation of the complement cascade (3).

In the classical complement pathway, IgG bound to antigen, typically displayed on bacterial membranes, recruits the C1 complex, which comprises the subcomponents C1q, C1r, and C1s. C1q has six globular domains tethered by a collagen-like stalk, which are directly involved in the interaction with the IgG Cγ2 domains (4). We have recently demonstrated that IgG molecules bound to antigens spontaneously assemble on the membrane, resulting in hexameric ring formation through the Fc region and thus facilitating avidity-driven binding to C1q (5).

We have carried out research focussing on a unique short-chain variant of mouse IgG2a(κ) that does not conform to this scenario. This variant, termed IgG2a(s), which was included in a panel of mouse anti-dansyl switch variant antibodies produced by hybridoma clones isolated by Herzenberg and co-workers (6), lacks the entire Cγ1 domain, resulting in a direct link between the V_H_ domain and the hinge segment (7). Notably, IgG2a(s) exhibits the ability to bind and thereby activate C1 even in the absence of an antigen (8, 9). Using high-speed atomic force microscopy (HS-AFM), we visualized that monomeric IgG2a(s) interacts with C1q in a 1:1 stoichiometry in the absence of antigen (10). Furthermore, our investigation has revealed that the mouse Cκ domain demonstrates a latent potential to bind to C1q. This discovery implies that, along with the conventional C1q binding site on the Fc region, their interactions are mediated by a secondary site within the Cκ domain, concealed under the presence of the Cγ1 domain.

This finding may hold implications for human pathology, as IgG variants with deletion of the Cγ1 domain are frequently observed in cases of gamma heavy chain disease (11). Furthermore, the potential C1q binding site of IgG could offer insights into the engineering of antibodies featuring innovative functionalities. With these insights as motivation, we endeavoured to uncover hidden C1 binding sites within the C_L_ domains of human and mouse antibodies using nuclear magnetic resonance (NMR) spectroscopy in conjunction with HS-AFM.

## Methods

### Complement components

Human C1 was purchased from Fitzgerald Industries International, Boston, MA, USA. C1q was purified from pooled human serum (Cosmo Bio CO., LTD, Tokyo, Japan) by two-step precipitation at low ionic strength as previously described (5).

### Expression and purification of C_L_ domains

A series of C_L_ domains, i.e. human Cκ, Cλ1, Cλ2, Cλ3, and Cλ7 and mouse Cκ, were bacterially expressed with a substitution of the C-terminal cysteine with serine followed by a hexahistidine tag. A gene encoding one of these C_L_ domains was subcloned into pET21a (Merck Millipore, Burlington, MA, USA) and expressed in *Escherichia coli* BL21-(DE3) (Agilent Technologies, Santa Cara, CA, USA). For NMR observation, these recombinant proteins were uniformly labelled with ^2^H, ^13^C, and/or ^15^N using an M9 medium containing 99.8% ^2^H_2_O, 2 g/l of [u-^13^C_6_]glucose, and/or 0.5 g/l of [^15^N]ammonium chloride for cell culture according to a previously described protocol (12). After sonication and centrifugation, the soluble fraction of the cell lysate was subjected to affinity chromatography with Ni^2+^-charged Chelating Sepharose (Cytiva, Tokyo, Japan). The proteins were further purified by size exclusion chromatography using a Superdex 75 pg column (Cytiva, Tokyo, Japan). For spectral assignments and 3D-structure determination, the proteins were treated with tobacco etch virus protease to eliminate the hexahistidine tag. The site-directed mutagenesis of human Cκ was performed using a mutagenesis kit (TOYOBO, Tokyo, Japan), and expression and purifications were carried out in the same way as above.

### NMR measurements

For all NMR measurements, the recombinant C_L_ domains were dissolved in 0.25 ml of 5 mM sodium phosphate buffer (pH 6.0) containing 50 mM NaCl and 5% (v/v) ^2^H_2_O. Conventional ^1^H–^15^N heteronuclear single quantum coherence (HSQC) spectra were measured using 100 μM ^15^N-labelled C_L_ domains. For spectral assignment and 3D-structure determination, a series of triple resonance experiments, HNCO, HN(CA)CO, HNCA, HN(CO)CA, HNCACB, CBCA(CO)NH, HBHA(CO)NH, HCCH-TOCSY, HCC(CO)NH, ^15^N-edited-NOESY, and ^13^C-edited-NOESY, along with^1^H–^15^N HSQC, were performed using uniformly ^13^C- and ^15^N-labelled C_L_ domains at a concentration of 1.5 mM. Amino acid selective labelling was also used for spectral assignments in an auxiliary manner (13). All NMR spectral data were acquired at 25°C using an AVANCE NEO 800 spectrometer with TCI CryoProbe and AVANCE III HD 800 spectrometer with TXI CryoProbe (Bruker BioSpin). Chemical shifts of ^1^H were referenced to DSS (0 ppm), while ^15^N and ^13^C chemical shifts were referenced indirectly using the gyromagnetic ratios of ^15^N, ^13^C, and ^1^H (γ^15^N/γ^1^H = 0.10132905, γ^13^C/γ^1^H = 0.25145020). For transferred cross-saturation (TCS) experiments (14), ^2^H- and ^15^N-labelled Cκ domain was dissolved at a concentration of 100 μM in the presence of 5 μM C1q and ^1^H–^15^N HSQC spectra were acquired with on-resonance (1 ppm) and off-resonance (40 ppm) proton irradiation.

Spectral data were analysed using Topspin and NMRtist software (15, 16). Molecular graphics were prepared using PyMOL (http://www.pymol.org/).

### HS-AFM observation

A circular mica substrate with a diameter of 1.5 mm (Furuuchi Chemical, Tokyo, Japan) was secured on a glass stage using acryl adhesive. First, a 2 μl droplet of 2 mM NiCl_2_ solution was placed onto a freshly cleaved mica substrate. Following a 3-min incubation, the Ni^2+^-coated mica substrate was rinsed with 100 μl of Milli-Q water. Then, a 1 μl droplet of hexahistidine-tagged C_L_ solution was placed on the mica substrate to immobilize through the Ni^2+^-histidine chelation. After a 3-min incubation, the sample was thoroughly washed by the observation [50 mM Tris-HCl (pH 8.0), 150 mM NaCl, and 2 mM CaCl_2_] buffer. Subsequently, a 2 μl droplet of C1/C1q in solution was placed onto the C_L_-covered mica substrate, followed by the sample being washed with the TNC buffer. All HS-AFM observations were carried out in the TNC buffer at 25°C using a laboratory-built HS-AFM operated in tapping mode (17, 18). We used small cantilevers (BL-AC7DS: Olympus, Tokyo, Japan) with a spring constant of ~0.2 Nm^−1^, a quality factor of approximately 2, and a resonant frequency of ~0.6 MHz in the solution. The AFM probe, which was made from amorphous carbon, was fabricated with electron-beam deposition using a scanning electron microscope (19, 20). To prevent interference with the interaction between the C_L_ and the C1/C1q, we carefully controlled the force applied by the AFM probe to the sample. Typically, the free oscillation amplitude of the cantilevers was adjusted to be 1–2 nm, and the amplitude set-point for feedback control was set to approximately 90% of the free oscillation value. The count of C1 or C1q within the designated scanning (200 × 200 nm^2^) was tallied following a 30-second observation period. For each specimen, analyses were conducted on 10 varied areas.

### Protein isoelectric point calculation

The protein isoelectric point was calculated using Compute pI/Mw (21).

## Results

### Bacterial expression of the recombinant C_L_ domains

In addition to the mouse Cκ domain, which is a component of IgG2a(s), our investigation also encompassed human C_L_ domains, which are classified into two types: Cκ and Cλ types. The Cλ type is divided into four distinct subtypes Cλ1, Cλ2, Cλ3, and Cλ7 (22). These domains were synthesized using bacterial production methods, incorporating serine substitution of the C-terminal cysteine residue to avoid disulfide-linked dimerization, C-terminal hexahistidine tagging for purification and HS-AFM observation, and stable isotope labelling for NMR observation. In order to assess their structural robustness, we performed an NMR spectroscopy on these recombinant proteins. The HSQC spectral data confirmed the structural integrity of all C_L_ domains, except the Cλ2 domain, which was largely unstructured when isolated in solution (Supplementary Fig. 1). Therefore, we decided to carry out further experiments on the remaining five C_L_ domains, i.e. human Cκ, Cλ1, Cλ3, and Cλ7 along with mouse Cκ.

### HS-AFM observation of the interaction of C_L_ with C1/C1q

**Figure 1.**
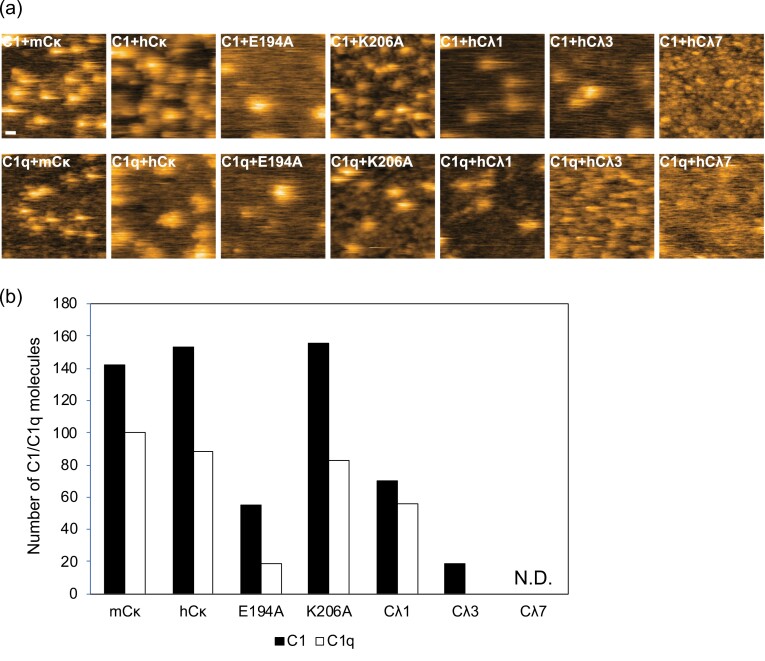
HS-AFM observation of the interaction of C_L_ domains with C1/C1q. (a) Typical HS-AFM images of C1 and C1q were observed on the C_L_-covered mica surface (indicated by the white arrows). Scale bar = 20 nm for all images. (b) The number of C1/C1q counted in 10 different scanning areas for each sample.

**Figure 2.**
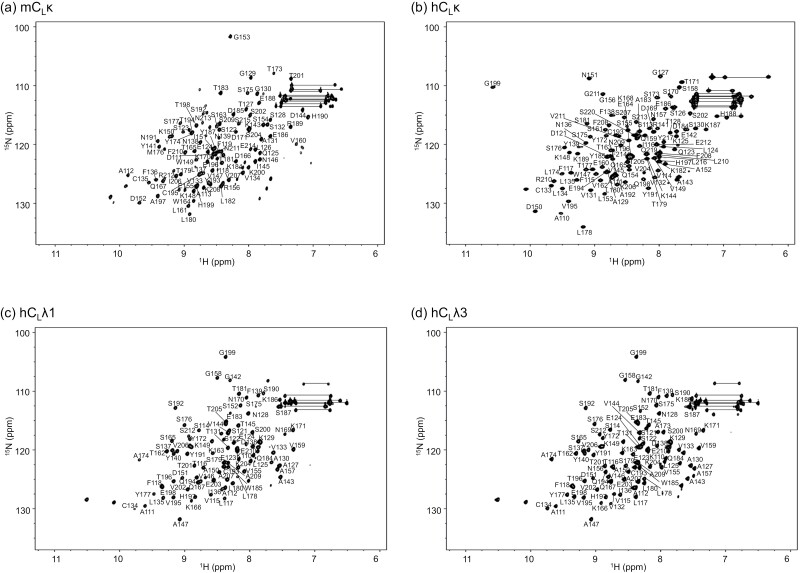
^1^H–^15^N HSQC spectral assignments of recombinant C_L_ domains. The ^1^H–^15^N HSQC spectra of uniformly ^13^C/^15^N-labelled C_L_ domains are displayed with backbone assignments for (a) mCκ, (b) hCκ, (c) hCλ1, and (d) hCλ3.

In a previous study using mouse Cκ, we established a protocol for quantitative characterization of the interaction between C_L_ and C1 (or C1q) on the basis of single-molecule HS-AFM observation (10). Using this protocol, we evaluated whether human C_L_ domains can interact with C1/C1q. We anchored each C_L_ domain onto a Ni^2+^-coated mica surface using the C-terminal hexahistidine tag and introduced a solution containing C1 or C1q to monitor their deposition on the surface. The results indicated that human Cκ exhibited comparable C1/C1q binding capabilities to mouse Cκ, which surpassed the Cλ types: within the human Cλ types, Cλ1 still had C1/C1q binding capabilities, whereas Cλ7 essentially did not exhibit binding (Fig. 1, Supplementary Fig. 2). The trend in binding preference to these C_L_ domains was similar between C1 and C1q; for each case, the accumulation of C1 was greater than that of C1q. We performed NMR spectral assignments and 3D structural determination for the C1-reactive C_L_ domains, i.e. mouse Cκ and human Cκ, Cλ1, and Cλ3; thereby confirming that they all adopt similar Ig folds in solution (Fig. 2 and Supplementary Fig. 3).

### TCS analysis to identify the C1q-binding site in C_L_

**Figure 3.**
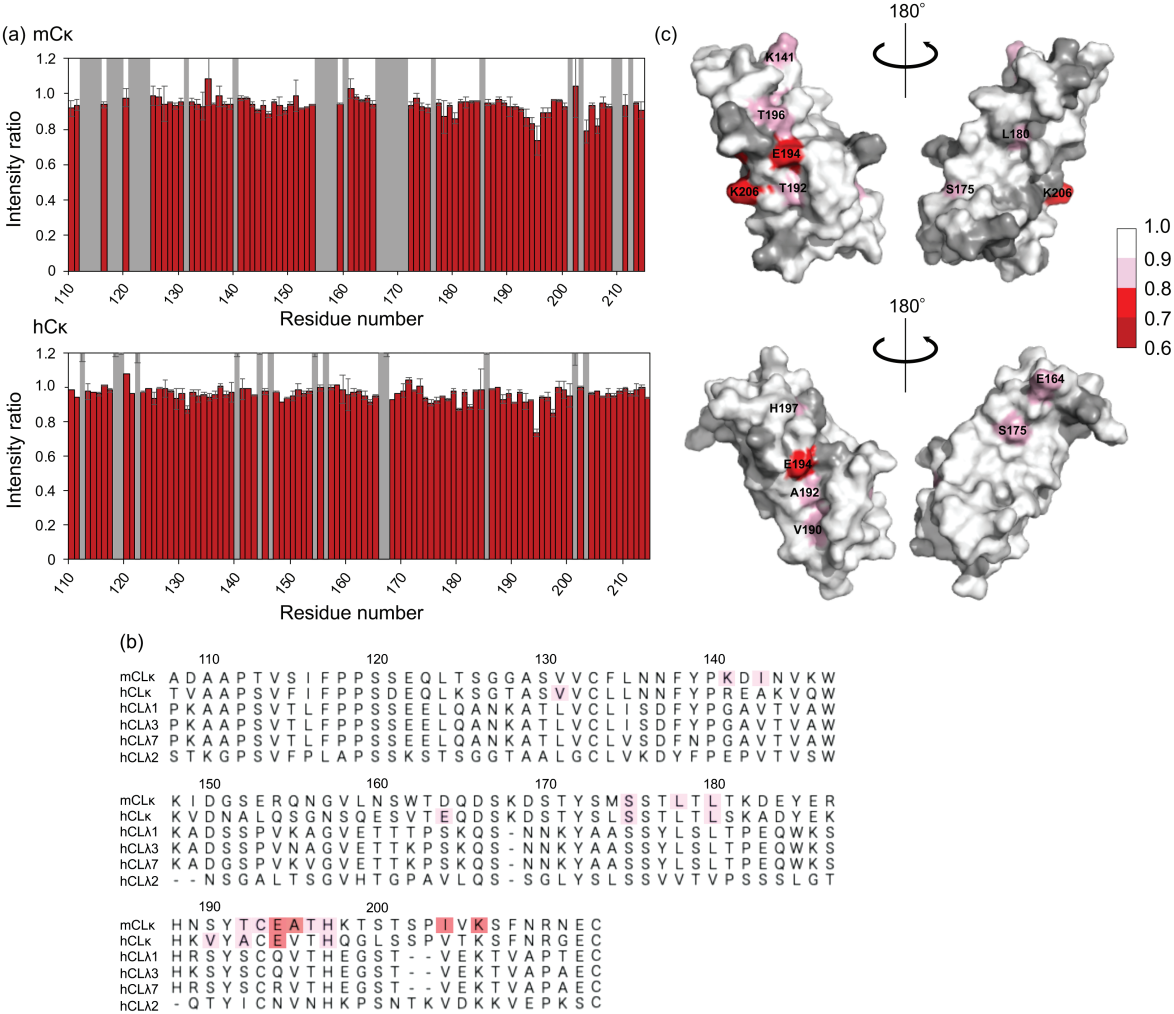
TCS analysis of the interaction between Cκ and C1q. (a) Plots of the intensity ratios of the backbone amide peaks originating from C_L_ with on-resonance and off-resonance irradiation of C1q protons. Proline residues and residues whose ^1^H–^15^N HSQC peaks could not be used as probes because of peak overlapping and/or broadening are shown by grey bars. The intensity ratios are shown as the mean ± SD of three independent experiments. The residues showing significant peak intensity reduction are mapped on (b) amino acid sequences and (c) NMR-derived 3D structures, with the magnitude of peak intensity attenuation due to the TCS effect indicated by a gradient scale.

We conducted TCS experiments for the human and mouse Cκ domains because they have a greater ability to interact with C1/C1q than the rest. We observed the attenuation of the intensity of ^1^H–^15^N HSQC peaks originating from the Cκ domains caused by saturation transferred from C1q, thus identifying their amino acid residues directly involved in the interaction with C1q (Fig. 3a). The amino acid residues that exhibited attenuation of peak intensity as a result of the TCS effect were mapped on NMR-derived 3D structural models as well as on amino acid sequences (Fig. 3b and c). The results indicate that the human and mouse Cκ domains share a common primary binding site for C1q, which is centred on Glu194, and other C1q contact residues are scattered on their surfaces, as typified by Lys206 in mouse Cκ, suggesting their multiple C1q-binding modes. We prepared E194A and K206A mutants of the human Cκ domain and examined their C1/C1q binding by HS-AFM. The E194A mutation compromised the C1/C1q binding capacity, while the K206A mutation did not (Fig. 1).

## Discussion

In our previous study on mouse IgG2a(s), we presented evidence indicating that the mouse Cκ domain has the capacity to bind to C1q (10). Although this interaction itself is weak, the avidity substantially enhances C1q binding as a result of simultaneous binding with the canonical C1q-binding site in Fc (Supplementary Fig. 5). In this investigation, we have extended our findings to demonstrate that the human Cκ domain also possesses C1q binding capabilities. However, Cλ domains, on the whole, exhibit a markedly reduced binding capacity. Our study identified Glu194 as a critical residue for C1q binding, a residue that is conserved in both mouse and human Cκ domains but conspicuously absent in Cλ domains. Notably, in Cλ7, Glu194 is replaced by arginine, rendering it incapable of binding to C1q. This observation strongly suggests that the presence of a negative charge at position 194 is a key determinant of C1q binding. However, this residue is exposed to solvent and accessible to C1q regardless of whether the Cγ1 domain is present or absent (Supplementary Fig. 3), which does not explain why IgG with Cγ1 does not spontaneously bind to C1q in the absence of antigen.

An intriguing aspect of our research is the discrepancy in the isoelectric points (pI) between Cκ and Cλ domains. Both the mouse and human Cκ domains have lower pI values, with pI = 5.6, while Cλ domains exhibit higher pI values, ranging from 6.9 to 9.1 for Cλ1 (6.9), Cλ2 (9.1), Cλ3 (6.9), and Cλ7 (8.5). This discrepancy suggests that electrostatic interactions play a critical role in binding to the positively charged C1q head (pI = 8.9) in a neutral solution. The structural instability observed in isolated Cλ2 may be attributed to its extremely high pI. Within the Fab region, other domains, i.e. V_H_, V_L_, and Cγ1, also have higher pI values of approximately 9, making them electrostatically repulsive to C1q. Consequently, the removal of the Cγ1 domain (pI = 9.1) facilitates the interaction of C1q with the Cκ domain in IgG. Consequently, the removal of the Cγ1 domain (pI = 9.1) facilitates the interaction of C1q with the Cκ domain in IgG. This interaction is mediated through the Glu194 site and other cryptic binding sites that were previously concealed by Cγ1 (Fig. 3c and Supplementary Fig. 4). Furthermore, deletion of the Cγ1 domain enhances motional freedom of the Cκ domain, enabling multiple modes of interaction with C1q. This results in simultaneous binding to the Fc and Cκ domains in the C_H_1-deleted IgG variant, thereby, leading to C1 activation even in the absence of an antigen. The importance of the motional freedom of the Cκ domain is also implicated in the fact that restricting the two Cκ domains in one IgG2a(s) molecule by disulfide linking results in loss of C1q-binding capability and subsequent failure of spontaneous C1 activation (9).

The C1 binding of Cκ resulting from the loss of C_H_1 potentially leads to uncontrolled activation of the complement pathway in the absence of antigen *in vivo*. This situation poses a risk, particularly in cases of gamma heavy chain disease. However, deliberate exploration of the potential C1q binding capabilities of Cκ offers exciting prospects in antibody engineering. One straightforward application is the incorporation of Cκ as a C1q-binding domain into various engineered antibody formats. To date, efforts have been made to improve complement-dependent cytotoxicity by introducing mutational changes into the Fc region, aimed at improving the direct C1q-binding site and promoting hexamer formation (23, 24). However, it is possible that modifying Cκ and its adjacent domains in the Fab region can lead to the creation of IgG antibodies that activate the complement cascade without the need for hexamer formation. It has been observed that antigen binding in the variable region can induce allosteric conformational changes in the Fab constant region (25). These findings suggest some potential development of monomeric antibodies that selectively bind to C1q and trigger the complement system in response to antigen binding, provided that suitable mutations are introduced to Cκ and Cγ1.

In pursuing this avenue of research, it is crucial to carefully consider the balance of charges within and between the Cκ domain and its neighbouring domains. For example, reducing the isoelectric point (pI) of Cγ1 may enhance the binding of IgG to C1q through its Cκ domain. Furthermore, the pI of the antibody domain plays a role in its interaction with serum proteins such as albumin, which can influence the interactions of IgG with effector functions (26). Consequently, future investigations should systematically explore charge modifications in IgG domains.

Traditionally, the prevailing understanding has been that IgG recognizes antigens through the Fab region and interacts with effector molecules via the Fc region. However, recent studies have begun to reshape this paradigm. In particular, it has been demonstrated that IgG can engage with FcγRIIIa through specific subsites within the Fab constant region, in addition to the established canonical site in the hinge-proximal Fc region (25, 27). On the other hand, this study underscores the presence of potential subsites in Fab for C1q binding. These subsites may serve as promising targets for antibody engineering aimed at controlling interactions with effector molecules for enhancing the performance of therapeutic antibodies.

## Supplementary Material

dxae017_suppl_Supplementary_Figures_S1-S5

## Data Availability

Assigned chemical shift data for mouse Cκ, human Cκ, human Cλ1, and human Cλ3 in solution were deposited in the BMRB under the accession numbers 36631, 36630, 36634, and 36633, respectively. Their 3D structures determined by NMR were deposited in PDB under the accession numbers 8XKK, 8XKJ, 8XLC, and 8XLB, respectively.
