# Supplementary material for: Identification of potential C1-binding sites in the immunoglobulin CL domains
Source: Int Immunol. 2024 Apr 2;36(8):405–12. doi: 10.1093/intimm/dxae017 (PMC11245854; doi:10.1093/intimm/dxae017)
Supplement: dxae017_suppl_Supplementary_Figures_S1-S5 [file dxae017_suppl_supplementary_figures_s1-s5.docx]

**Identification of potential C1-binding sites in the immunoglobulin C_L_ domains**

Saeko Yanaka^1,2,3^, Atsuji Kodama^1^, Shigetaka Nishiguchi^1^, Rina Fujita^1^, Jiana Shen^1,2^, Pornthip Boonsri^4^, Duckyong Sung^1,2^, Yukiko Isono^1^, Hirokazu Yagi^1,2^, Yohei Miyanoiri^5^, Takayuki Uchihashi^1,6^, and Koichi Kato^1,2,*^

*^1^ Exploratory Research Center on Life and Living Systems (ExCELLS), Institute for Molecular Science (IMS), National Institutes of Natural Sciences, 5-1 Higashiyama, Myodaiji, Okazaki 444-8787, Japan*

*^2^ Faculty and Graduate School of Pharmaceutical Sciences, Nagoya City University, 3-1 Tanabe-dori, Mizuho-ku, Nagoya 467-8603, Japan*

*^3^ Graduate School of Pharmaceutical Sciences, Kyushu University, 3-1-1 Maidashi, Higashi, Fukuoka 812-8582, Japan*

*^4^ Department of Chemistry, Faculty of Science, Srinakharinwirot University, Bangkok 10110, Thailand*

*^5^ Institute for Protein Research, Osaka University, 3-2 Yamadaoka, Suita, Osaka, 565-0871, Japan*

*^6^ Department of Physics and Institute for Glyco-core Research (iGCORE), Nagoya University, Furocho, Chikusa, Nagoya, 464-8602 Aichi, Japan*

*^*^* ***Correspondence to K. Kato****, kkatonmr@ims.ac.jp*

**
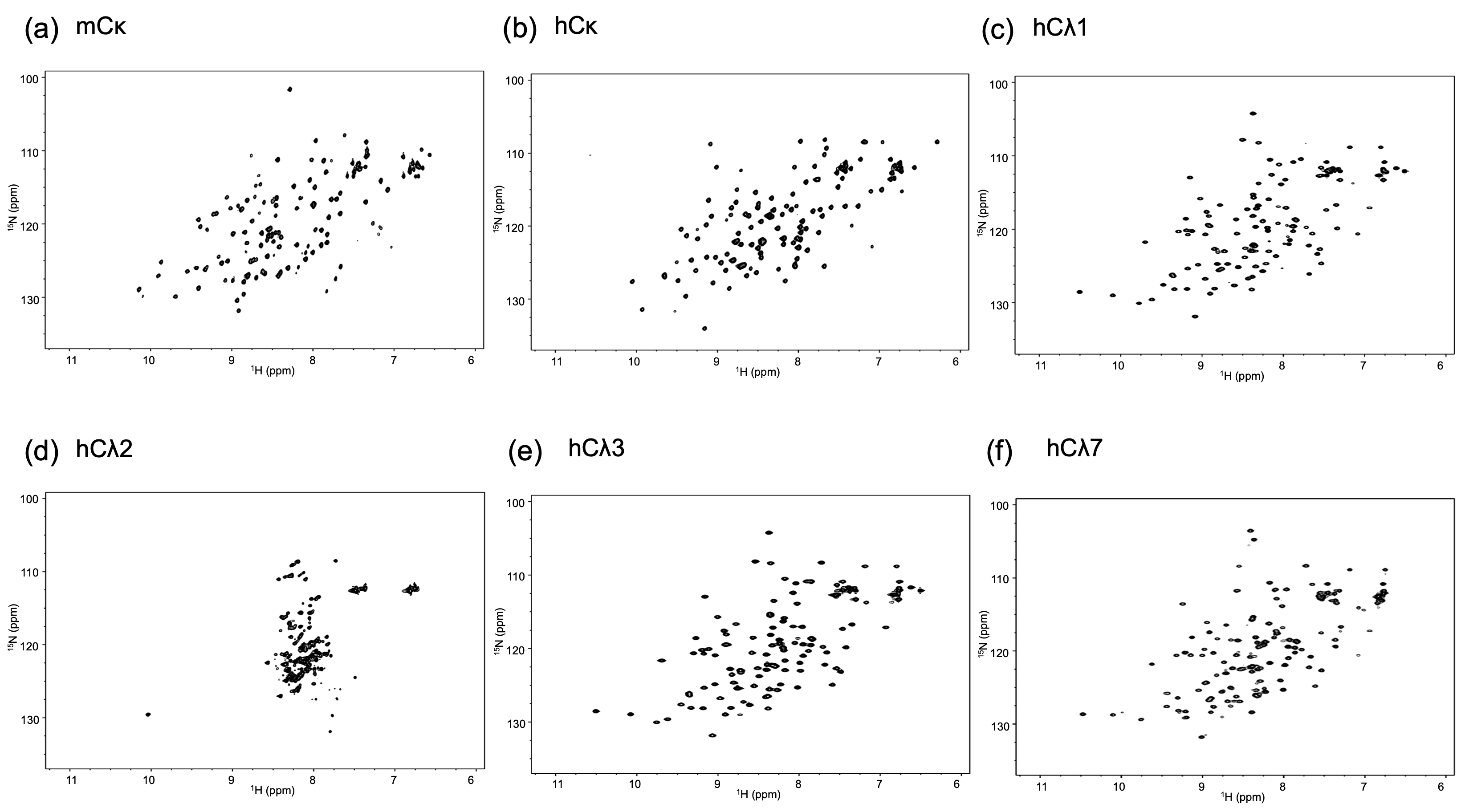
**

**Supplementary Figure 1: ^1^H-^15^N HSQC spectra of C_L_ domains.**

The ^1^H-^15^N HSQC spectra of ^15^N-labeled C_L_ with a hexahistidine tag are displayed for (a) mCκ, (b) hCκ, (c) hCλ1, (d) hCλ2, (e) hCλ3, and (f) hCλ7.


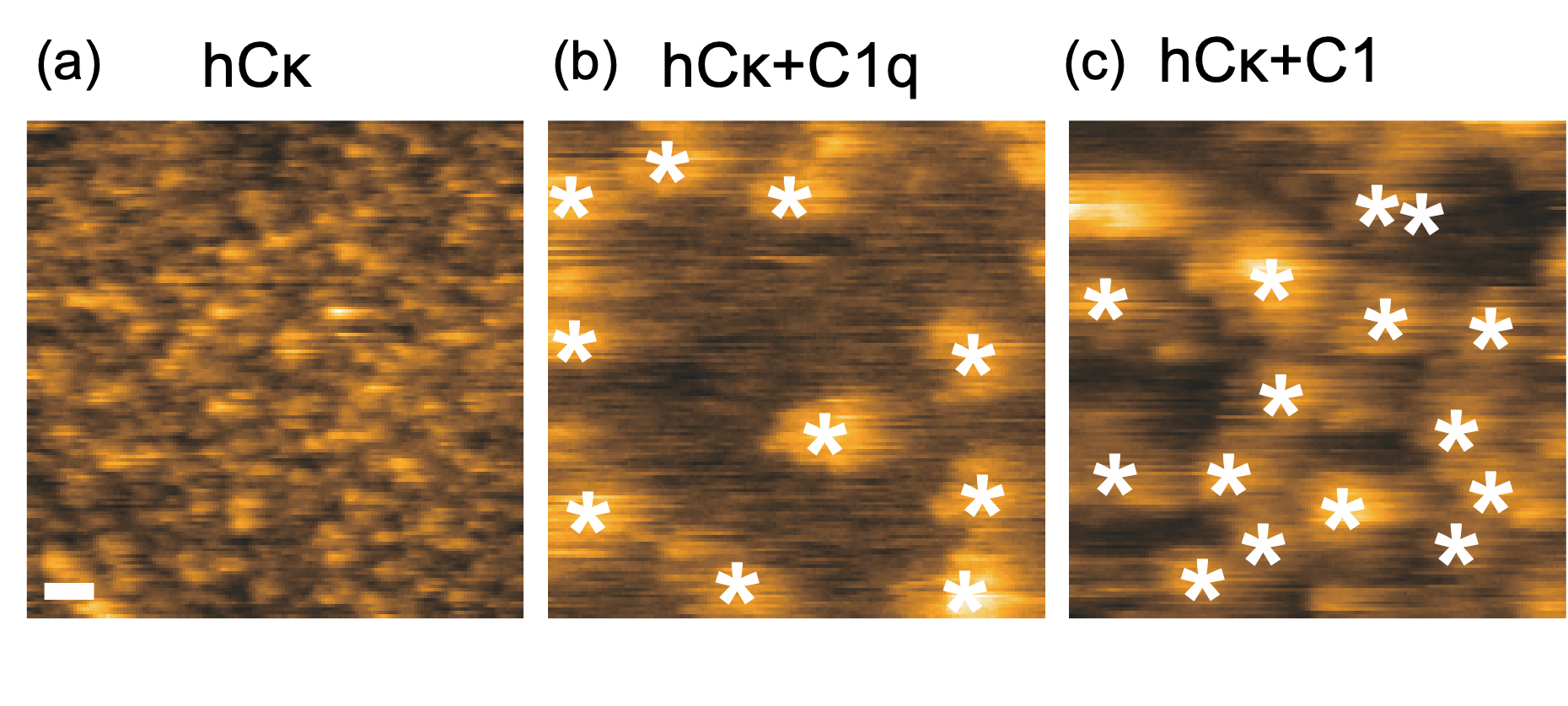


**Supplementary Figure 2: Representative HS-AFM images depicting the C_L_ domains with and without interacting C1/C1q.**

(a) immobilized hCκ domains visualized as discreet small particles densely covering the mica surface, (b) C1q or (c) C1 manifesting as larger particles marked with asterisks. Scale bar = 20 nm for all images.


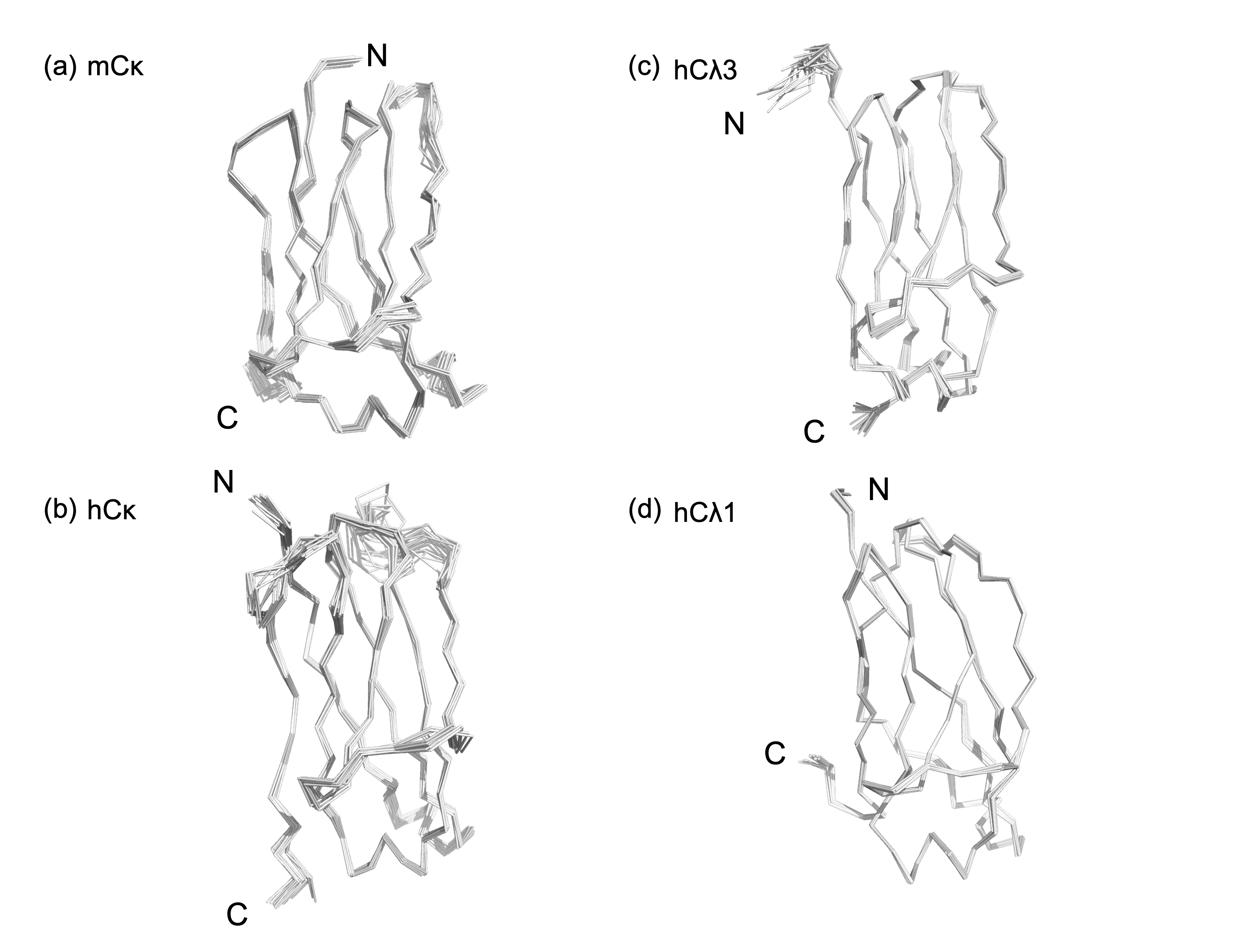


**Supplementary Figure 3: 3D structures of C_L_ domains determined by NMR.**

Converged structures of (a) mCκ, (b) hCκ, (c) hCλ1, and (d) hCλ3. Structural models are illustrated as an ensemble of the final 20 lowest energy structures.


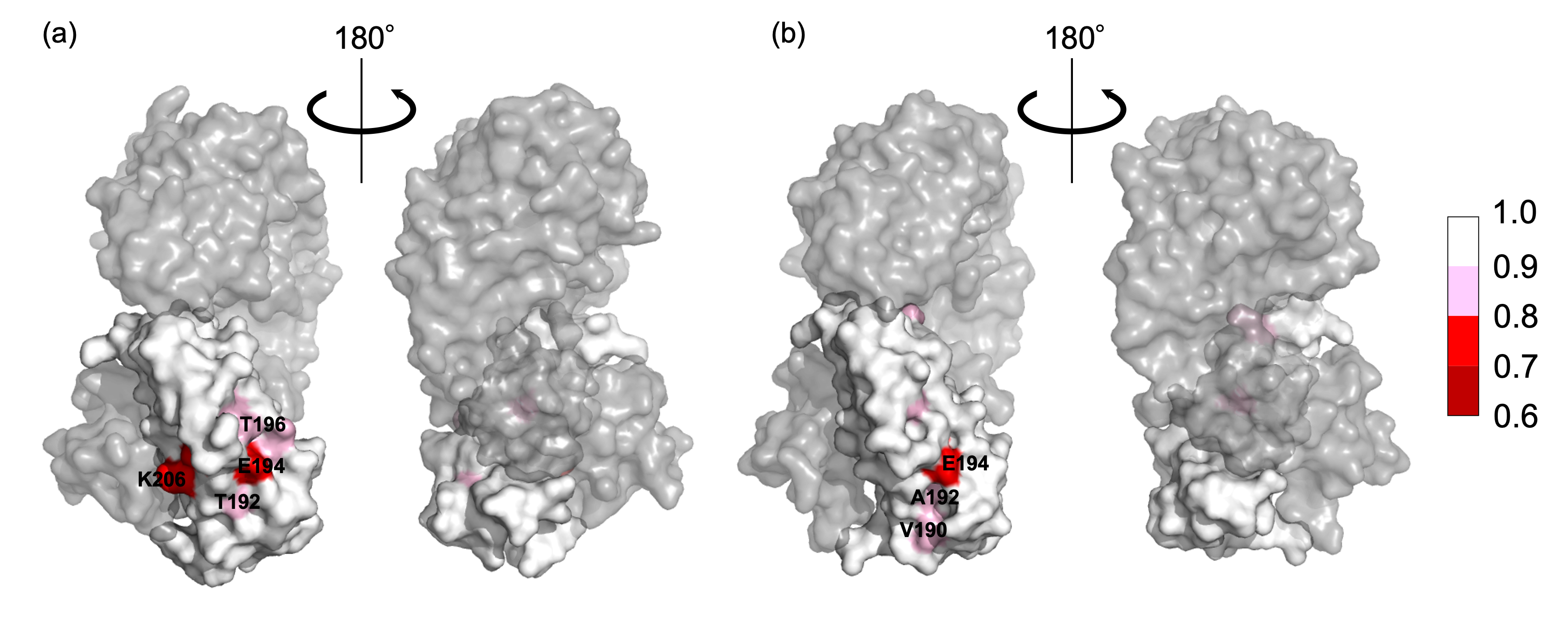


**Supplementary Figure 4: Mapping of TCS effects on Fab crystal structures.**

The residues showing significant peak intensity reduction in TCS analysis are mapped on crystal structures of (a) mouse Fab (PDBID:1AIF) and (b) human Fab (PDBID: 1VGE) with the color gradient as in Figure 3c.


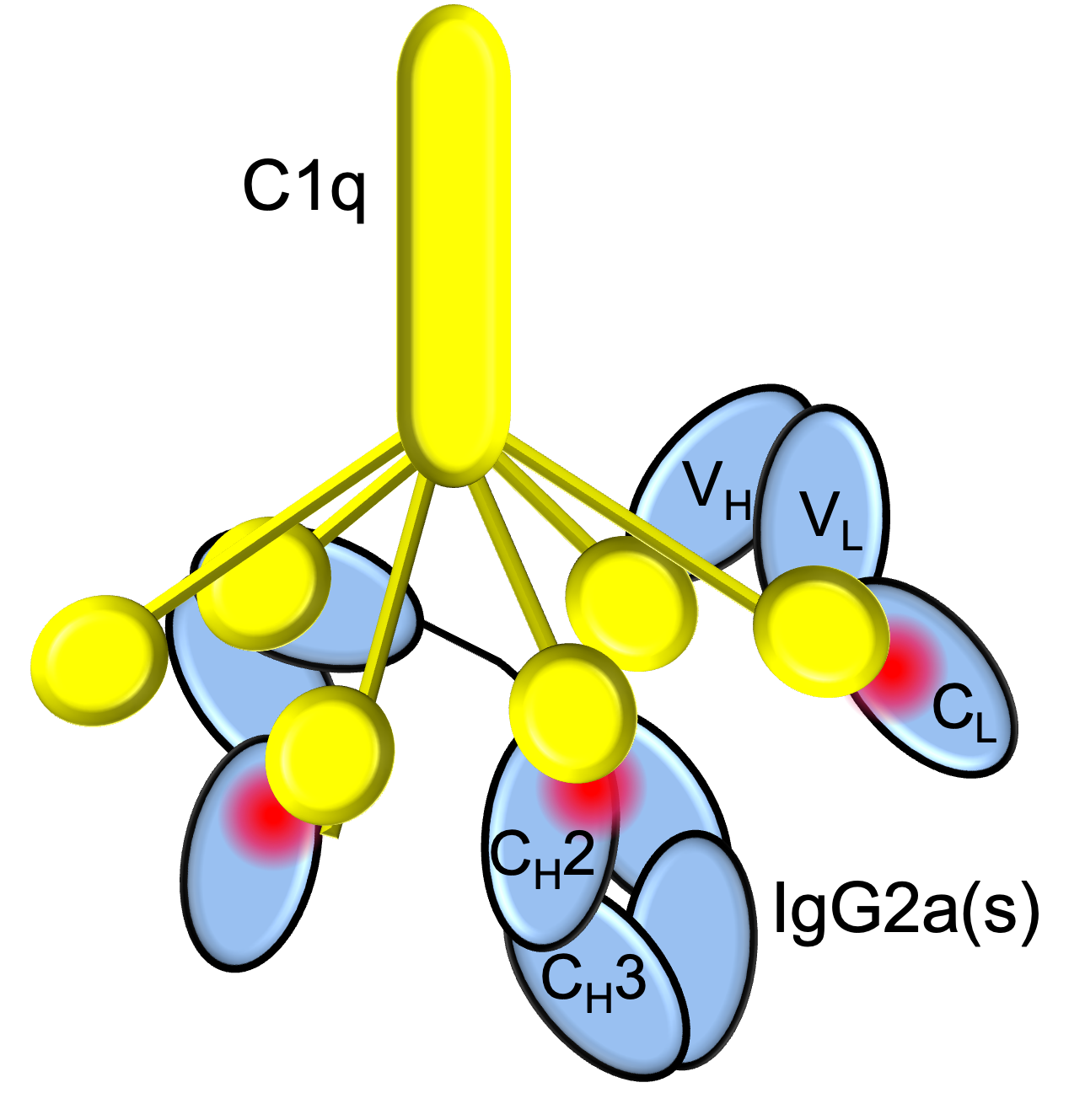


**Supplementary Figure 5: Schematic representation illustrating the hypothetical multivalent interaction mode between C1q and mouse IgG2a(s).**
